# Type and etiology of liver cirrhosis are not related to the presence of hepatic encephalopathy or health-related quality of life: a cross-sectional study

**DOI:** 10.1186/1471-230X-8-46

**Published:** 2008-10-15

**Authors:** Evangelos Kalaitzakis, Axel Josefsson, Einar Björnsson

**Affiliations:** 1 Section of Gastroenterology and Hepatology, Department of Internal Medicine, Sahlgrenska University Hospital, Gothenburg, Sweden

## Abstract

**Background:**

Hepatic encephalopathy has a negative impact on health-related quality of life (QoL) in liver cirrhosis. There are scarce and conflicting data on whether type or etiology of liver cirrhosis could be related to hepatic encephalopathy in patients with cirrhosis. We aimed to determine the impact of cirrhosis etiology on hepatic encephalopathy and whether hepatic encephalopathy affects health-related QoL among patients with cirrhosis of different etiologies.

**Methods:**

A total of 156 cirrhotic patients were prospectively evaluated for the presence of hepatic encephalopathy according to the West-Haven criteria as well as by means of two psychometric tests. Patients with cryptogenic cirrhosis or cirrhosis due to mixed hepatocellular/cholestatic etiologies were excluded. Fasting plasma glucose levels were also measured. QoL was evaluated by means of a validated questionnaire (SF-36).

**Results:**

Diabetes mellitus was more common in patients with hepatocellular cirrhosis compared to those with cholestatic cirrhosis but the two groups did not differ in cirrhosis severity or the prevalence of hepatic encephalopathy (p > 0.05). The groups of patients with cirrhosis due to alcohol, hepatitis C, or cholestatic liver disease did not differ in severity of liver cirrhosis or the prevalence of hepatic encephalopathy (p > 0.05). Patients with cirrhosis of different etiologies did not differ in any SF-36 domain (p > 0.05). In multivariate analysis, performance at neuropsychological testing was independently related only to age, diabetes mellitus, and the Child-Pugh score whereas the SF-36 physical component summary only to the Child-Pugh score and hepatic encephalopathy.

**Conclusion:**

Cirrhosis etiology does not seem to be related to hepatic encephalopathy or health-related QoL. Cognitive impairment is associated mainly with age, liver disease severity and diabetes mellitus.

## Background

Patients with liver cirrhosis are prone to develop cognitive dysfunction termed hepatic encephalopathy (HE). The clinical manifestations of HE range from subtle intellectual and personality changes, that can be detected only by means of neuropsychological and neurophysiological tests (i.e. minimal HE), to coma [1]. The pathogenesis of HE is incompletely understood but ammonia is considered to play a central role [1,2].

Published data on cognitive impairment in cirrhosis due to cholestatic liver disease compared to cirrhosis due to hepatocellular disease are few and conflicting [3,4]. Recently diabetes mellitus was found to be associated with HE in hepatitis C cirrhosis [5] and with performance at neuropsychological cognitive testing in an unselected cirrhotic population [6]. The prevalence of diabetes mellitus is known to vary according to the etiology of cirrhosis, being higher in patients with cirrhosis due to hepatitis C or alcoholic liver disease (hepatocellular disease) compared to patients with cirrhosis due to cholestatic liver disease [7]. However, it is unknown whether any potential difference in the prevalence of HE between cholestatic and hepatocellular cirrhosis can be explained by the different prevalence of diabetes mellitus in these groups of patients.

Previous studies have explored cognitive function in alcoholic patients as well as in patients with chronic hepatitis C and primary biliary cirrhosis (PBC), and all have been reported to have cognitive impairment in the absence of cirrhosis [8-12]. However few investigations have assessed the importance of etiology of liver disease for cognitive function in cirrhosis [13-15] and data on the potential effect of liver disease etiology on minimal or overt HE diagnosed according to currently accepted criteria [16] are scarce.

Health-related quality of life (QoL) is impaired in patients with cirrhosis [17-20]. Liver disease severity [17,18,20] and hepatic encephalopathy [17,21] are known to have a negative impact on QoL in this group of patients. Although most studies have shown that the etiology of cirrhosis does not have any major effect on QoL [17,19,20], published data are not unanimous as patients with cholestatic cirrhosis have been reported to have less impairment in QoL than patients with hepatocellular cirrhosis [18]. Furthermore, it is unknown whether potential differences in the prevalence of hepatic encephalopathy in patients with cirrhosis of different etiologies are reflected in health-related QoL.

The primary aim of the current study was to investigate the relation of the type (hepatocellular vs. cholestatic) and etiology of liver cirrhosis with the presence of hepatic encephalopathy. We also aimed to evaluate the relation of etiology of liver disease with health-related QoL (particularly in view of potential differences in the prevalence of hepatic encephalopathy among groups of cirrhotic patients with different etiologies).

## Methods

### Patients

Consecutive adult patients with liver cirrhosis admitted to the gastroenterology ward or attending the outpatient clinic of the gastroenterology department at a transplant center in Sweden were prospectively enrolled. Inclusion criterion was liver cirrhosis of any cause. The diagnosis of liver cirrhosis was established histologically or based on the presence of at least 2 of the following: characteristic imaging features, esophageal or gastric varices, ascites, increased international normalized ratio (INR) that could not be attributed to any other cause. Patients unable to understand Swedish as well as those unable to give written informed consent or to fill in questionnaires (e.g. due to severe comorbidities such as dementia and psychosis, or debilitating hepatic encephalopathy) were excluded. Also excluded were patients with cryptogenic cirrhosis, mixed hepatocellular and cholestatic chronic liver disease (e.g. overlap syndrome), or with cirrhosis after liver transplantation. Patients with cryptogenic cirrhosis were excluded as the underlying etiology is unclear. Although a history of alcohol abuse is lacking in these patients it is difficult to exclude with certainty an alcoholic etiology in some. It has been recognized, in recent years, that a significant proportion of patients with cryptogenic cirrhosis might be due to a long-standing non-alcoholic steatohepatitis (NASH). Patients with NASH cirrhosis were included in the current study only if NASH had been appropriately diagnosed prior to cirrhosis development. Patients with unknown cirrhosis etiology despite extensive investigations and in the absence of a history of diagnosed NASH prior to cirrhosis development were excluded to allow proper allocation of enrolled patients to type and etiology groups. Out of 176 consecutive patients who fulfilled the inclusion criteria and were approached, 156 patients (87%) agreed to participate in the study and completed the questionnaires. Patients hospitalized because of acute diseases or complications related to liver disease were evaluated when stable clinical conditions were reached. No patient was on dialysis and none had hepatorenal syndrome. Patient data were collected from medical records, including etiology of liver disease, previous variceal bleeding, existing esophageal or fundic varices, and hepatocellular carcinoma. No patient was receiving interferon therapy at inclusion in the study. Serum albumin and bilirubin as well as fasting plasma glucose and the INR were measured by standard in-house methods. Patients were considered having diabetes if they were receiving antidiabetic treatment (oral hypoglycemic agents or insulin) or had elevated fasting plasma glucose levels (> 7 mmol/l). The severity of the liver disease was assessed according to the Child-Pugh classification and the Model for end-stage liver disease (MELD) score [22]. The presence of ascites was evaluated by means of abdominal ultrasonography upon inclusion in the study. Patient files were carefully scrutinized in order to ascertain the grounds on which the diagnosis of the etiology of cirrhosis was established. Patients with cirrhosis due to alcoholic liver disease, viral or autoimmune hepatitis, or NASH were designated as the group with hepatocellular cirrhosis whereas patients with PBC or primary sclerosing cholangitis (PSC) were designated as the cholestatic group. The study protocol was approved by the ethics committee of the University of Gothenburg and all patients gave written informed consent before participation.

### Evaluation of hepatic encephalopathy

HE was evaluated according to the guidelines proposed by the 11^th ^World Congress of Gastroenterology in 1998 [16]. It was graded clinically on a scale from 0 to 4 according to the West-Haven criteria and cognitive function was also assessed (on the same day) by means of two psychometric tests the number connection test A and B (NCT-A/B) [16]. Instructions for administration of NCT-A and B were strictly followed [23]. Every effort was undertaken to rule out concomitant neurologic disease (such as cerebrovascular events, subdural hematoma, Wernicke's disease, drug intoxication) as indicated clinically. The results of NCT-A and B of each patient were compared to age-corrected normal values obtained from the general population [23]. On the same day as clinical evaluation for HE and administration of NCT-A and B were performed, fasting venous blood samples were drawn from every patient for plasma ammonium ion measurement which was performed directly according to a standard in-house method. Minimal hepatic encephalopathy was defined as absence of overt hepatic encephalopathy assessed by the West-haven criteria and NCT-A > 3 standard deviations (SD) and/or NCT-B > 3SD of the general population [16,23].

### Assessment of health-related quality of life

The generic health-related QoL instrument Short Form-36 (SF-36) was developed as a comprehensive measure of general health status for use in the Medical Outcomes Study, and has been thoroughly tested for validity and reliability [24-27]. This questionnaire assesses the extent to which an individual's health limits physical, emotional, and social functioning. It consists of 36 items organised in eight domains: physical functioning, role limitations caused by physical health problems, bodily pain, general health perceptions, vitality, social functioning, role limitations caused by emotional problems and mental health. The SF-36 is scored from 0 to 100, with higher scores indicating better health-related QoL. Two comprehensive indices of health-related QoL can also be computed: physical component summary, summarizing the first four domains, and mental component summary, summarizing the last four domains. SF-36 has previously been used for the assessment of QoL in patients with chronic liver disease [17,19-21]. Normative data from the Swedish general population are available, as well as thorough assessment of validity and reliability of the Swedish version of SF-36 [27].

### Statistics

Data are expressed as mean (SD). The student's t-test or the Mann-Whitney test was performed as appropriate in order to compare continuous variables. The Chi-square or the Fisher's exact test was used as appropriate for comparisons between categorical variables. The Pearson's correlation coefficient was calculated for correlation analysis between continuous variables. For multiple comparisons among groups one-way ANOVA for continuous variables or chi-square test for categorical variables was performed. If p < 0.05 the Bonferroni test (continuous variables) or the chi-square test (categorical variables) was used for post-hoc comparisons. First, patients with different types of liver disease (hepatocellular vs. cholestatic) were compared as regards to the presence of hepatic encephalopathy as well as other clinical parameters. Subsequently, patients with one of the major three etiologies (alcoholic cirrhosis vs. cirrhosis due to hepatitis C vs. cholestatic cirrhosis) were compared. In an attempt to explore independent predictors of NCT-A and NCT-B performance times as well as the SF-36 physical component summary, all parameters that were univariately correlated at p < 0.1 with these variables, were entered into stepwise linear multiple regression analyses. All tests were two-tailed and conducted at a 5% significance level.

## Results

A total of 29 patients had cholestatic cirrhosis (11 with PBC and 18 with PSC) and 127 had hepatocellular cirrhosis (55 alcoholic; 37 viral (32 hepatitis C, 5 hepatitis B); 24 mixed alcoholic and viral; 6 autoimmune hepatitis; 4 NASH; one alpha-1-antithrypsine deficiency). Seventeen out of 79 patients with alcoholic or mixed alcoholic cirrhosis admitted to active alcohol overconsumption. The rest claimed to have been abstinent during the previous 3–6 months but only 28 these were followed-up by a specialized addiction center and had documented abstinence (by means of random urine or breath tests).

### Hepatic encephalopathy in hepatocellular vs. cholestatic cirrhosis

Diabetes mellitus was less common among patients with cholestatic cirrhosis compared to those with hepatocellular cirrhosis but the two groups did not differ significantly in the prevalence of (minimal or overt) hepatic encephalopathy nor in the prevalence of other complications or in severity of liver cirrhosis (table 1). Patients with PBC vs. PSC did not differ significantly in the prevalence of (minimal or overt) HE or diabetes mellitus nor in the severity of liver cirrhosis (data not shown). However, there are significant age and gender differences between patients with PBC and PSC (data not shown) rendering these comparisons hard to interpret. Within the group of patients with alcoholic or mixed alcoholic and viral cirrhosis, the prevalence of HE did not differ between patients actively drinking and patients with documented abstinence from alcohol (data not shown).

**Table 1:** Demographic and clinical characteristics in patients with hepatocellular and cholestatic liver cirrhosis

	Hepatocellular cirrhosis (n = 127)	Cholestatic cirrhosis (n = 29)	p-value
Age	56 (11)	54 (14)	0.496
Female/Male	33/94 (26%/74%)	9/20 (31%/69%)	0.580
Outpatients/inpatients	104/23 (82%/18%)	25/4 (86%/14%)	0.579
Previous variceal bleeding	37 (29%)	8 (28%)	0.953
Esophageal and/or fundic varices	88 (69%)	24 (83%)	0.139
Ascites	55 (43%)	9 (31%)	0.225
Hepatocellular carcinoma	22 (17%)	1 (3.4%)	0.06
Number connection test A			
Mean (SD) (sec)	61 (44)	50 (44)	0.258
Above 3 SD^a^	17 (13%)	3 (10%)	0.567
Number Connection test B			
Mean (SD) (sec)	148 (82)	118 (69)	0.08
Above 3 SD^a^	26 (20%)	4 (14%)	0.303
Overt hepatic encephalopathy	32 (25%)	4 (14%)	0.216
(West-Haven)^b^			
Grade I	30 (23.5%)	3 (10.5%)	
Grade II	2 (1.5%)	1 (3.5%)	
Minimal hepatic encephalopathy^c^	9 (7%)	2 (7%)	0.93
Hepatic encephalopathy	41 (32%)	6 (21%)	0.175
(minimal or overt)			
Fasting plasma ammonium ion (μmol/l)	58 (37)	52 (21)	0.292
MELD score	13.7 (6.2)	14.3 (5.4)	0.645
Child-Pugh score	8.6 (2.4)	8.8 (2)	0.752
Diabetes Mellitus	44 (35%)	3 (10%)	0.01

Data are presented as mean (SD) or n (%) as appropriate

^a ^Compared to age-corrected normal values from the general population

^b ^Overt hepatic encephalopathy according to West-Haven criteria: none with grade III or IV

^c ^Defined as absence of overt hepatic encephalopathy and number connection test A > 3SD and/or number connection test B > 3SD

Patients with compared to those without diabetes mellitus took longer time to perform NCT-A and B (table 2). However, the two groups did not differ significantly in the prevalence of (minimal or overt) hepatic encephalopathy nor in the severity or prevalence of complications of liver cirrhosis with the exception of hepatocellular carcinoma that was more common among patients with diabetes (table 2). When patients with hepatocellular and cholestatic cirrhosis were analyzed separately, diabetes mellitus was not related to the prevalence of (minimal or overt) HE in either group (data not shown).

**Table 2:** Demographic and clinical characteristics in patients with and without diabetes mellitus

	With Diabetes (n = 47)	Without Diabetes (n = 109)	p-value
Age	58 (12)	54 (11)	0.055
Female/Male	8/39 (17%/83%)	34/75 (31%/69%)	0.07
Outpatients/inpatients	41/6 (87%/13%)	88/21 (81%/19%)	0.325
Previous variceal bleeding	11 (23%)	34 (32%)	0.309
Esophageal and/or fundic varices	30 (77%)	82 (79%)	0.804
Ascites	19 (40%)	45 (41%)	0.920
Hepatocellular carcinoma	11 (23%)	12 (11%)	0.045
Number connection test A			
Mean (SD) (sec)	73 (62)	53 (33)	0.012
Above 3 SD^a^	9 (19%)	11 (10%)	0.122
Number Connection test B			
Mean (SD) (sec)	164 (93)	132 (72)	0.035
Above 3 SD^a^	10 (21%)	20 (18%)	0.726
Overt hepatic encephalopathy	14 (30%)	22 (20%)	0.202
(West-Haven)^b^			
Grade I	12 (25.5%)	21 (19%)	
Grade II	2 (4.5%)	1 (1%)	
Minimal hepatic encephalopathy^c^	3 (6%)	8 (7%)	0.83
Hepatic encephalopathy	17 (36%)	30 (27%)	0.274
(minimal or overt)			
Fasting plasma ammonium ion (μmol/l)	60 (37)	55 (34)	0.433
MELD score	13.7 (6.5)	13.9 (5.8)	0.895
Child-Pugh score	8.6 (2.2)	8.7 (2.4)	0.883

Data are presented as mean (SD) or n (%) as appropriate

^a ^Compared to age-corrected normal values from the general population

^b ^Overt hepatic encephalopathy according to West-Haven criteria: none with grade III or IV

^c ^Defined as absence of overt hepatic encephalopathy and number connection test A > 3SD and/or number connection test B > 3SD

### Hepatic encephalopathy in alcoholic cirrhosis vs. cirrhosis due to hepatitis C vs. cholestatic cirrhosis

The group of patients with hepatocellular cirrhosis was heterogeneous. In order to explore the potential effects of specific etiologies of liver disease on hepatic encephalopathy, patients with mixed etiologies or with etiologies represented in low numbers in the current cohort (i.e. hepatitis B, NASH, autoimmune hepatitis, and alpha 1-antithrypsine deficiency) were excluded from further analysis. Subsequently, the groups of patients with cirrhosis due to alcoholic liver disease, hepatitis C, or cholestatic liver disease were compared to one another (table 3). Diabetes mellitus was less common in the group of cholestatic cirrhosis, but the three groups did not differ in severity of liver disease or the prevalence of (minimal or overt) hepatic encephalopathy (table 3).

**Table 3:** Demographic and clinical characteristics in patients with cirrhosis due to alcoholic liver disease, hepatitis C, and cholestatic liver disease

	Alcoholic liver cirrhosis (n = 55)	Hepatitis C cirrhosis (n = 32)	Cholestatic cirrhosis (n = 29)
Age	60 (8) *	54 (7)	54 (14)
Female/Male	12/43 (22%/78%)	9/23 (28%/72%)	9/20 (31%/69%)
Outpatients/inpatients	42/13 (76%/24%)	27/5 (84%/16%)	25/4 (86%/14%)
Previous variceal bleeding	15 (27%)	7 (22%)	8 (28%)
Esophageal and/or fundic varices	37 (67%)	21 (66%)	24 (83%)
Ascites	31 (56%)*	10 (31%)	9 (31%)
Hepatocellular carcinoma	6 (11%)	9 (28%)*	1 (3.4%)
Number connection test A			
Mean (SD) (sec)	68 (51)	52 (33)	50 (44)
Above 3 SD^a^	10 (18%)	2 (6%)	3 (10%)
Number Connection test B			
Mean (SD) (sec)	165 (87)	141 (83)	118 (69)
Above 3 SD^a^	13 (24%)	5 (16%)	4 (14%)
Overt hepatic encephalopathy	15 (27%)	6 (19%)	4 (14%)
(West-Haven)^b^			
Grade I	14 (25.5%)	6 (19%)	3 (10.5%)
Grade II	1 (2%)	0	1 (3.5%)
Minimal hepatic	4 (7%)	2 (6%)	2 (7%)
encephalopathy^c^	19 (34%)	8 (25%)	6 (21%)
Hepatic encephalopathy			
(minimal or overt)			
Fasting plasma ammonium ion (μmol/l)	50 (26)	51 (35)	52 (21)
MELD score	14.9 (7.2)	12.6 (5)	14.3 (5.4)
Child-Pugh score	9 (2.6)	8.2 (2.2)	8.8 (2)
Diabetes mellitus	20 (36%)	11 (34%)	3 (10%)*

Data are presented as mean (SD) or n (%) as appropriate

^a ^Compared to age-corrected normal values from the general population

^b ^Overt hepatic encephalopathy according to West-Haven criteria: none with grade III or IV

^c ^Defined as absence of overt hepatic encephalopathy and number connection test A > 3SD and/or number connection test B > 3SD

For comparisons of continuous variables if one-Way ANOVA was significant (p < 0.05), the post-hoc Bonferroni test was used for comparisons among different groups.

For comparisons of if the chi-square test was significant (p < 0.05) when data from all groups were tested, the chi-square test was used for post-hoc analysis among different groups

*p < 0.05 compared to the other two groups

### Number connection tests A and B performance times

Clinical parameters univariately related at p < 0.1 with the time needed to perform NCT-A or NCT-B were entered into stepwise multivariate regression analyses with NCT-A and NCT-B performance times as the dependent variables (table 4). The time needed to perform NCT-A was independently related to the Child-Pugh score and diabetes mellitus whereas the time needed to perform NCT-B was independently related only to the Child-Pugh score and age (table 4).

**Table 4:** Factors independently correlated to the time needed to perform number connection test A and B after multivariate analysis in patients with liver cirrhosis (n = 156)

	Adjusted R^2 ^% (for whole model)	Unstandardized beta coefficient
Time needed to perform NCT-A	20.3	
Child-Pugh score		6.37**
Diabetes mellitus		16.9*
Time needed to perform NCT-B	29.9	
Child-Pugh score		12.4**
Age (per year)		2.84**

* p < 0.05, **p < 0.001

### Etiology of cirrhosis and health-related QoL

**Figure 1:**
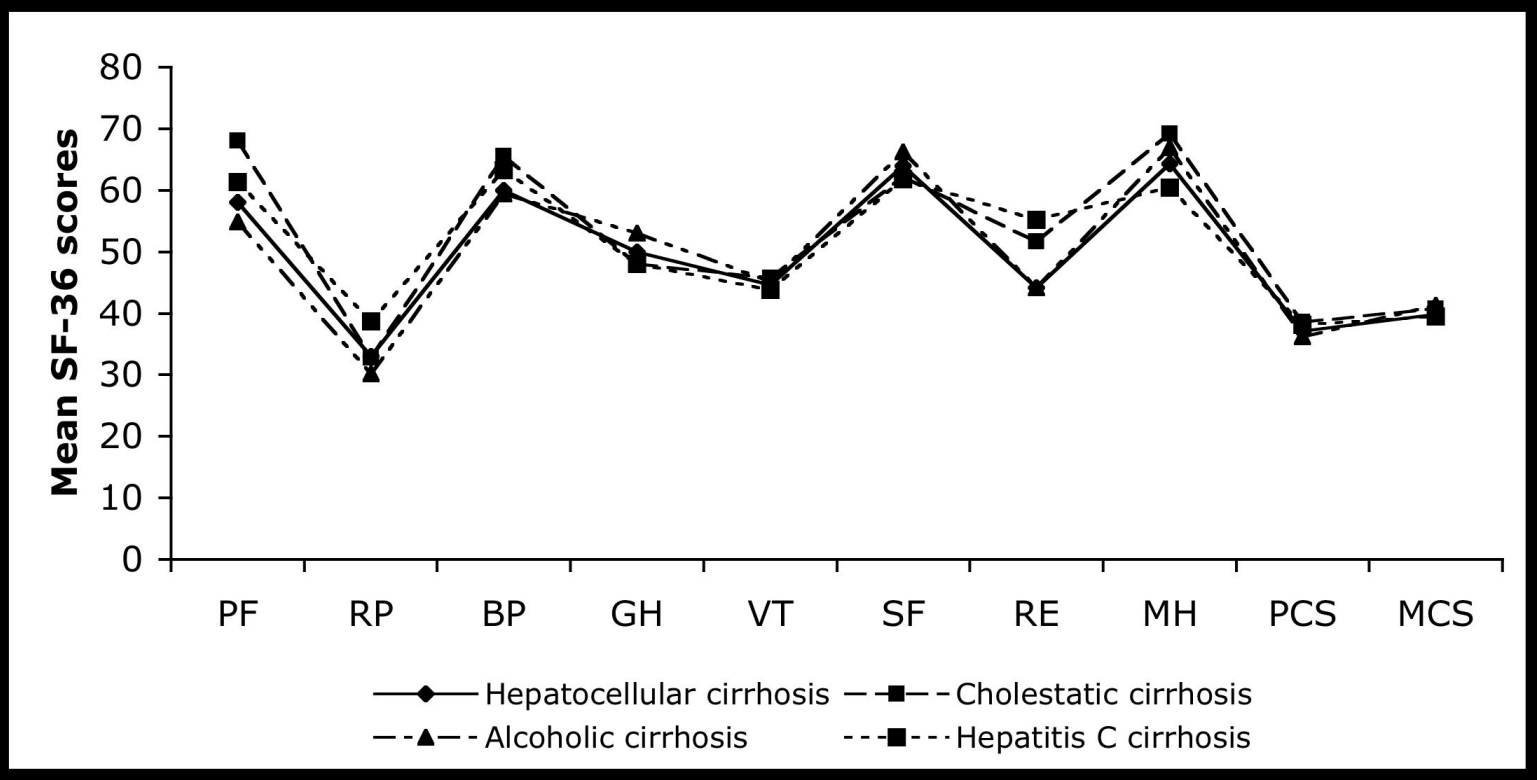
**Health-related quality of life assessed as mean SF-36 domain and summary scores in patients with liver cirrhosis with different etiologies (totally n = 156)**. Mean SF-36 domain and summary scores did not differ significantly among different groups (p > 0.05 for all). PF, physical functioning; RP, role limitations caused by physical health problems; BP, bodily pain; GH, general health perceptions; VT, vitality; SF, social functioning; RE, role limitations caused by emotional problems; MH, mental health; PCS, physical component summary; MCS, mental component summary.

Patients with compared to those without (minimal or overt) HE had lower SF-36 physical component summary (30.1 (12.8) vs. 40.6 (10.8), p < 0.001) and mental component summary (36.4 (12.8) vs. 41.8 (14.4), p < 0.05). However, etiology of liver cirrhosis was not related to health-related QoL (figure 1). All parameters in table 3 that were univariately related to the SF-36 physical component summary at p < 0.1 were entered into a stepwise regression analysis. Only the Child-Pugh score (unstandardized beta coefficient = -1.36, p = 0.01) and HE (unstandardized beta coefficient = -3.59, p = 0.012) were independently related to the SF-36 physical component summary.

## Discussion

In the current study, the etiology of liver cirrhosis was not related to HE or health-related QoL. Diabetes mellitus was more common among patients with hepatocellular cirrhosis compared to those with cholestatic cirrhosis, in accordance to published data [7], and neither cirrhosis severity nor the prevalence of HE differed between the two groups. Furthermore, the prevalence of HE did not differ among the groups of cirrhosis due to alcohol, chronic hepatitis C, or cholestatic liver disease. To our knowledge, this is the first study to investigate the impact of etiology of cirrhosis on minimal and overt HE, as assessed according to currently accepted criteria [16], with simultaneous assessment of diabetes mellitus and measurement of plasma ammonia levels.

These findings are in line with previous reports on the relation of cognitive impairment with the etiology of liver disease [4,13,14,28]. Despite initial reports that cognitive impairment detected by means of neuropsychological testing varied according to the type -hepatocellular vs. cholestatic- of cirrhosis [3], a subsequent study demonstrated that cerebral computed tomography scan abnormalities were similarly common in both hepatocellular and cholestatic liver disease [4]. A recent investigation showed that cognitive dysfunction was frequent in PBC patients and unrelated to the severity of liver disease [12]. However, few of the patients included in this study had frank cirrhosis and patients with PBC were not compared with controls with another chronic liver disease [12]. Therefore, these findings may not be extrapolated to cirrhotic populations due to PBC or PSC.

In previous imaging studies cerebral abnormalities have been detected with computed tomography in non-alcoholic cirrhotic patients [29] as well as with magnetic resonance imaging in cirrhotic patients irrespective of liver disease etiology [30]. Although cirrhotic alcoholics have been reported to exhibit more gross pathology than non-alcoholic cirrhosis on ratings of cerebral atrophy assessed by computed tomography in another imaging study, the authors noted that the two groups were more similar to each other than they were different on planimetric measurements [31]. Cognitive impairment has been observed in non-cirrhotic patients with alcoholism [8] and with chronic hepatitis C [10,11,32]. However, in some hepatitis C cohorts cognitive decline has not been shown to differ from that of patients with chronic liver disease of other etiologies [10,32]. Liver disease severity is thought to be the main determinant of cognitive dysfunction in alcoholic cirrhosis [13,14,28] although some controversy exists [33]. In a recent study in patients with cirrhosis without overt HE (according to the West Haven criteria), minimal HE was proposed to be more common in cirrhotic patients with hepatitis C [34]. However, this difference was only observed in univariate analysis of data and was not tested in multivariate analysis [34]. Apart from this report data comparing cognitive function of patients with hepatitis C cirrhosis with that of patients with cirrhosis due to other etiologies are largely lacking. Our findings support previously published data that alcoholic etiology is not a major determinant of cognitive dysfunction in cirrhotic patients [13,14,28] and further indicate that neither hepatitis C nor cholestatic liver disease have a major effect on hepatic encephalopathy in cirrhosis.

Diabetes mellitus has been shown to be associated to HE in hepatitis C cirrhosis [5]. Insulin resistance, which is common in cirrhotic patients, is also related to plasma ammonia levels [6]. We did observe an independent relation of diabetes mellitus with the time needed to perform NCT-A, in accordance with a previous report [6], but diabetes did not affect the prevalence of HE in our patient cohort. The same was true when the potential effect of diabetes on HE was analyzed separately in the subgroups of hepatocellular or cholestatic cirrhosis. Although our study was not designed to assess the impact of diabetes on HE our results indicate that factors other than diabetes mellitus might be more important in determining HE in cirrhosis of various etiologies.

Most previous studies have not detected any difference in health-related QoL indices among patients with liver cirrhosis of different etiologies [17,19,20]. Younossi et al compared health-related QoL in patients with hepatocellular and cholestatic cirrhosis and found that physical dimensions of QoL were less impaired in patients with cholestatic disease than in those with hepatocellular disease [18]. We found that health-related QoL was related to hepatic encephalopathy in line with previously published data [17,21] but we did not observe any significant differences in any QoL dimension between hepatocellular and cholestatic cirrhosis. These apparently different findings may be explained by the different patient groups recruited in the two studies. In the study of Younossi et al the group of hepatocellular cirrhosis consisted mainly of patients with alcoholic and cryptogenic cirrhosis as well as patients with autoimmune hepatitis [18]. On the other hand, patients with cryptogenic cirrhosis were excluded from the current investigation and 39/127 patients with hepatocellular disease had viral cirrhosis. Furthermore, in the previous study the proportion of patients with Child-Pugh C cirrhosis was 3.3% in the cholestatic and 26.6% in the hepatocellular group [18] whereas there were not any significant differences in cirrhosis severity between the two groups in the current study. Our findings are in accordance with previously published data indicating HE and cirrhosis severity are important determinants of QoL in cirrhosis whereas the etiology of liver disease does not seem to play a major role [17,20,21].

Certain methodological limitations of the currents study should be taken into consideration. First, HE was evaluated by means of clinical assessment and psychometric tests but no quantitative neurophysiologic tools (such as electroencephalography) were used. Although neurophysiologic tools are often used in research studies there is no clear consensus as to the validity of these tests when used alone or in combination [35]. In the current study, the guidelines of the 11^th ^World Congress of Gastroenterology were followed in the evaluation of HE [16]; clinical assessment and simple bedside psychometric tests were chosen as they are easily applied in everyday practice. Second, although in the current study no major differences were observed in demographic variables or in severity of liver disease among groups of patients with different etiologies, ideally patients in the different groups should be matched for age, gender, and severity of liver disease. Third, the current study is a cross-sectional one. Thus, a cause-effect relationship between the measured variables would be hard to establish. Last, although our investigation is the largest, to date, exploring the relation of the type of liver cirrhosis (cholestatic vs. hepatocellular) with the presence of hepatic encephalopathy and although all patients with cholestatic cirrhosis under our care were asked to participate, a type-II error cannot be excluded. Further multicenter studies might be necessary to fully delineate the potential role of the type and etiology of cirrhosis in hepatic encephalopathy.

## Conclusion

We conclude that the etiology of liver disease is not related to hepatic encephalopathy or health-related QoL in liver cirrhosis. Performance at psychometric testing is affected by age, diabetes mellitus, and cirrhosis severity. Although diabetes mellitus was more common among patients with hepatocellular compared to those with cholestatic cirrhosis, the prevalence of HE did not differ between the two groups.

## Abbreviations

HE: hepatic encephalopathy; PBC: primary biliary cirrhosis; QoL: quality of life; INR: international normalized ratio; NASH: non-alcoholic steatohepatitis; MELD: model for end-stage liver disease; PSC: primary sclerosing cholangitis; NCT: number connection test; SD: standard deviation; SF-36: Short Form-36.

## Competing interests

The authors declare that they have no competing interests.

## Authors' contributions

EK participated in the design of the study, in data collection, analysis and interpretation, and wrote the manuscript. AJ participated in the design of the study and data collection/analysis, and revised critical the manuscript critically. EB participated in the design of the study and the interpretation of the results and revised the manuscript critically. All authors read and approved the final manuscript.

## Pre-publication history

The pre-publication history for this paper can be accessed here:


